# Can Only the Shape Feature in Radiomics Help Machine Learning Show That Bladder Cancer Has Invaded Muscles?

**DOI:** 10.7759/cureus.45488

**Published:** 2023-09-18

**Authors:** Harun Özdemir, Sena Azamat, Merve Sam Özdemir

**Affiliations:** 1 Department of Urology, Başakşehir Çam and Sakura City Hospital, Istanbul, TUR; 2 Department of Radiology, Başakşehir Çam and Sakura City Hospital, Istanbul, TUR

**Keywords:** muscle invazion, shape feature, cancer staging, bladder cancer, urogenital neoplasms

## Abstract

Objectives: The presence of muscle invasion is an important factor in establishing a treatment strategy for bladder cancer (BCa). The aim of this study is to reveal the diagnostic performance of radiomic shape features in predicting muscle-invasive BCa.

Methods: In this study, 60 patients with histologically proven BCa who underwent a preoperative MRI were retrospectively recruited. The whole tumor volume was segmented on apparent diffusion coefficient (ADC) maps and T2W images. Afterward, the shape features of the volume of interest were extracted using PyRadiomics. Machine learning classification was performed using statistically different shape features in MATLAB^®^ (The MathWorks, Inc., Natick, Massachusetts, United States).

Results: The findings revealed that 27 bladder cancer patients had muscle invasion, while 33 had superficial bladder cancer (53 men and seven women; mean age: 62±14). Surface area, volume, and relevant features were significantly greater in the invasive group than in the non-invasive group based on the ADC maps (P<0.05). Superficial bladder cancer had a more spherical form compared to invasive bladder cancer (P=0.05) with both imaging modalities. Flatness and elongation did not differ significantly between groups with either modality (P>0.05). Logistic regression had the highest accuracy of 83.3% (sensitivity 82.8%, specificity 84%) in assessing invasion based on the shape features of ADC maps, while K-nearest neighbors had the highest accuracy of 78.2% (sensitivity 79.1%, specificity 69.4%) in assessing invasion based on T2W images.

Conclusions: Shape features can be helpful in predicting muscle invasion in bladder cancer using machine learning methods.

## Introduction

Bladder cancer (BCa) is the most common cancer of the urinary system, accounting for approximately 420,000 new cases and 160,000 deaths per year in the United States [1]. BCa can be divided into several types, including urothelial carcinoma, squamous epithelial carcinoma, and adenocarcinoma; more than 90% of cases are urothelial carcinoma [2]. Urothelial cell carcinomas are divided into low-grade and high-grade cancers according to pathologic assessment and are also classified as muscle-invasive and non-muscle-invasive cancers.

Identification of the muscle-invasion status of BCa is critical for making treatment decisions. In 2018, the Vesical Imaging Reporting and Data System (VI-RADS) criteria were published to standardize the clinical use and reporting of multiparametric magnetic resonance imaging (mp-MRI) in bladder cancer. The scoring of BCa in the VI-RADS system focuses on the differentiation of superficial and muscle-invasive cancers [3,4]. mp-MRI combining morphological T2-weighted (T2W) and diffusion-weighted imaging (DWI) and dynamic contrast imaging (DCE) has received attention due to its ability to improve the success of conventional MRI in the diagnosis and local staging of BCa [5].

Radiomics is defined as the transformation of images into higher-dimensional data, followed by the mining of these data to enhance decision-making support [6]. Among the extracted features are those dependent on the lesion's shape. These are descriptors of the region of interest, including its three-dimensional size and shape, and, unlike other radiomic data, they are independent of the gray-level intensity distribution, allowing for potentially greater reproducibility [7]. Shape features can also be evaluated visually without segmentation. In the literature, radiomics features have been used to distinguish between muscle-invasive and non-muscle-invasive types of BCa [8]. In this study, we aimed to use the radiomics shape features to predict muscle-invasive BCa and to evaluate its diagnostic performance in predicting muscle invasion with machine learning when only shape features are used.

## Materials and methods

Patient selection

A total of 60 patients with histopathologically proven BCa who underwent a preoperative MRI in Başakşehir Çam and Sakura City Hospital, Istanbul, Türkiye, from January 2020 to August 2022 were retrospectively reviewed. The following exclusion criteria were used: 1) incomplete MRI sequences and inadequate image quality due to magnetic or motion artifacts; 2) MR imaging that was not based on VI-RADS imaging guidelines [4]; 3) a history of surgery or treatment before MRI; 4) patients with no index lesion on MRI; 5) transurethral resection (TUR) specimens without smooth muscle layers; 6) patients having variant histology. The index lesion was regarded as the one with the highest VI-RADS score or, in the case of multiple high-scoring lesions, the one with the largest size. The research procedure was approved by the Ethics Committee of the Başakşehir Çam and Sakura City Hospital, and informed consent from patients was waived due to the study’s retrospective nature.

MRI acquisition

All examinations were performed on a 3.0 T MRI scanner (Philips Achieva; Koninklijke Philips N.V., Amsterdam, Netherlands). Patients were placed in a supine position, and a phased-array body coil was used for the imaging. Patients were instructed not to urinate for two hours after reaching the hospital in order to achieve sufficient bladder distension. Those who did not follow these rules were advised to drink at least 500 mL of water and then wait an hour before being assessed. The MRI protocols included the following sequences: T2WI (TR/TE: 4690 ms/119 ms; matrix: 400 ×256; field of view (FOV): 230 mm x230 mm) and T1C (TR/TE 3.8 ms/1.2 ms; matrix: 192 × 192; FOV: 270 mm x270 mm). A slice thickness of 2-4 mm and a gap of 0-0.4 mm were applied in all images. T1C images were acquired after administration of 0.2 ml/kg of gadobenate dimeglumine (Gd-BOPTA) (MultiHance®; Bracco S.p.A., Milan, Italy). DWI was acquired with spin-echo echo-planar imaging sequence according to the following parameters: TR/TE: 2500-5300 ms/61 ms, matrix: 128 × 128, FOV: 320 mm × 320 mm, slice thickness: 45 mm, slice gap: 0.3-0.4 mm, and three b values (b = 0.800 and 1000 s/mm^2^). Apparent diffusion coefficient (ADC) maps were automatically generated with a mono-axial decay model using the three b values.

Volume of interest delineation and texture analysis

**Figure 1:**
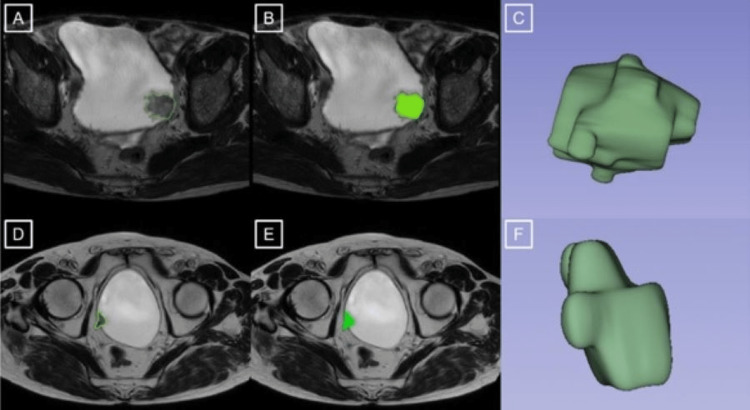
An example of image annotation performed on T2W images. Lesions on the bladder lateral wall (A,B,D,E) are segmented 3D images (C,F). The lesion in A, B, and C appears to have a rounder shape and a larger surface area. The pathology report indicated a high-grade non-invasive tumor. In D, E, F, it is seen that the shape of the lesion is flatter and the surface area is narrower. The pathology report indicates a high-grade invasive tumor.

The same urogenital radiologist defined index lesions with a polygonal region of interest (ROI) on each slice of the T2WI and ADC maps using 3D Slicer version 4.8.1 (http://slicer.org/). The extracted volumes of interest on the T2W-MRI and ADC maps were then evaluated using an open-source tool for extracting quantitative data from medical images (Pyradiomics, v2.1.2) (Figure 1). Thirteen shape features were recorded from the original images. The definitions of shape features are summarized in Table 1. Because these features are unaffected by the gray-level intensity distribution in the ROI, they can only be estimated on the original image and segmentation mask.

**Table 1:** The definitions of shape features

Shape Feature Name	Definition
Elongation	Elongation depicts the connection between the two largest principal components in the ROI shape.
Flatness	Flatness depicts the connection between the ROI shape’s greatest and smallest principal components.
Least Axis Length	The smallest axis length of the VOI-enclosing ellipsoid is recorded as the least axis length.
Major Axis Length	The largest axis length of the VOI-enclosing ellipsoid is recorded as the major axis length.
Maximum 2D Diameter Column	The largest pairwise Euclidean distance between the vertices of the tumor surface mesh in the coronal plane is recorded as the maximum 2D diameter.
Maximum 2D Diameter Row	The largest pairwise Euclidean distance between the vertices of the tumor surface mesh in the sagittal plane is recorded as the maximum 2D diameter.
Maximum 2D Diameter Slice	The largest pairwise Euclidean distance between the vertices of the tumor surface mesh in the axial plane is recorded as the maximum 2D diameter.
Maximum 3D Diameter	The largest pairwise Euclidean distance between tumor surface mesh vertices is recorded as the maximum 3D diameter.
Minor Axis Length	The second-largest axis length of the VOI-enclosing ellipsoid is recorded as the minor axis length.
Sphericity	Sphericity is an assessment of the roundness of the tumor region.
Surface Area	The surface area of each triangle in the mesh is calculated as the sum of all calculated sub-areas.
Surface Volume Ratio	A lower number corresponds to a more compact (sphere-like) form.
Voxel Volume	The volume of the VOI is estimated by multiplying the number of voxels in the VOI by the volume of a single voxel.

Statistical analysis

Data were normalized using min-max normalization. A Mann-Whitney U test was employed to assess the shape feature differences with each imaging modality between the muscle-invasive and non-invasive groups. A p-value <0.05 was considered statistically significant. Only features found to be significant in the statistical analysis were entered into the machine learning algorithms. Thirty different machine learning algorithms were run to classify muscle-invasive and non-invasive groups for validation of the statistical differences. The algorithm with the highest accuracy was selected for each modality. Leave-one-out cross-validation was used to evaluate the performance of the classification algorithms in terms of accuracy, sensitivity, and specificity. MATLAB Statistics and Machine Learning Toolbox R2019b (The MathWorks, Inc., Natick, Massachusetts, United States) were used for the statistical and machine learning analyses.

## Results

The study cohort consisted of 60 patients: 53 male (88%) and seven female (12%). The demographic features of the study cohort are presented in Table 2. Twenty-seven BCa patients showed muscle invasion, while 33 had non-muscle-invasive BCa. Surface area, volume, and relevant features, including least, major, and minor axis lengths, maximum 2D diameter row, and maximum 3D diameters, were significantly higher in the invasive group than in the non-invasive group (P<0.05). Statistical analysis of the shape features showed that non-muscle-invasive BCa (normalized=0.607±0.293) had more sphericity compared to invasive BCa (normalized=0.434±0.250). The surface volume ratio, flatness, elongation, and maximum 2D diameter slice and column did not differ significantly between the two groups in T2W images (P>0.05) (Table 3).

**Table 2:** Demographic features of the study cohort M: male; F: female

	Invasive	Non-invasive
Age (years), mean ± SD	63.7±13.9	60.7±15.6
Gender Distribution (M:F)	22:5	31:2
Number of Patients (n)	27	33

**Table 3:** Statistical differences in shape features based on T2W-MRI between the muscle-invasive and non-invasive groups

	Muscle Invasion	Muscle Non-invasion	P-value
Surface Volume Ratio	0.407±0.234	0.317±0.259	0.081
Sphericity	0.434±0.250	0.607±0.293	0.025
Flatness	0.513±0.278	0.476±0.285	0.779
Voxel Volume	0.178±0.197	0.096±0.219	<0.001
Surface Area	0.355±0.217	0.114±0.210	<0.001
Elongation	0.591±0.260	0.534±0.275	0.450
Least Axis Length	0.449±0.207	0.276±0.232	0.0017
Major Axis Length	0.538±0.210	0.249±0.243	<0.001
Minor Axis Length	0.539±0.214	0.294±0.254	<0.001
Maximum 2D Diameter Column	0.346±0.184	0.279±0.255	0.153
Maximum 2D Diameter Row	0.569±0.224	0.229±0.235	<0.001
Maximum 2D Diameter Slice	0.411±0.201	0.348±0.275	0.273
Maximum 3D Diameter	0.425±0.200	0.279±0.253	0.005

Of all the variables obtained from ADC maps, only elongation and flatness were in statistically similar ranges for the groups (P>0.05). However, the surface volume ratio, surface area, volume, and other relevant features, including least, major, and minor axis lengths, maximum 2D diameters, and 3D diameter, were significantly higher in the muscle-invasive group (P<0.05). Non-muscle invasive BCa (normalized=0.601±0.275) had a statistically more spherical form than muscle-invasive BCa (normalized=0.429±0.237) in the ADC maps, as seen in the T2W images (Table 4). Logistic regression had the highest accuracy of 83.3% (sensitivity=82.8% and specificity=84%) in assessing invasion based on the shape features of ADC maps, while K-nearest neighbors had the highest accuracy of 78.2% (sensitivity 79.1% and specificity 69.4%) in assessing invasion based on the shape features of T2W imaging (Table 5).

**Table 4:** Statistical differences in shape features based on ADC maps between the muscle-invasive and non-invasive groups ADC: apparent diffusion coefficient

	Muscle Invasion	Muscle Non-Invasion	P-Value
Surface Volume Ratio	0.424±0.241	0.131±0.181	<0.001
Sphericity	0.429±0.237	0.601±0.275	0.016
Flatness	0.448±0.259	0.589±0.270	0.060
Voxel Volume	0.191±0.198	0.086±0.205	<0.001
Surface Area	0.370±0.244	0.116±0.211	<0.001
Elongation	0.599±0.271	0.669±0.222	0.291
Least Axis Length	0.449±0.232	0.274±0.225	0.002
Major Axis Length	0.573±0.208	0.241±0.221	<0.001
Minor Axis Length	0.551±0.211	0.336±0.249	<0.001
Maximum 2D Diameter Column	0.515±0.196	0.286±0.266	<0.001
Maximum 2D Diameter Row	0.528±0.215	0.251±0.224	<0.001
Maximum 2D Diameter Slice	0.564±0.196	0.286±0.266	<0.001
Maximum 3D Diameter	0.597±0.190	0.278±0.245	<0.001

**Table 5:** The classification accuracy/sensitivity/specificity results based on shape features obtained from ADC maps and T2W images ADC: apparent diffusion coefficient; LOOCV: leave-one-out cross-validation; CV: cross validation

	Accuracy (%)	Sensitivity (%)	Specificity (%)	Features(#)	CV fold
Logistic Regression Shape Features Based on ADC Maps	83.3	82.8	84.0	11	LOOCV
K-Nearest Neighbors Shape Features Based on T2W	78.2	82.7	73.0	8	LOOCV

## Discussion

Radiomics is an emerging image analysis framework that provides more details than conventional methods. The extracted imaging features have been shown to reveal visually imperceptible information, extending beyond radiology to histopathology. In the literature, there are BCa radiomics studies employing MRI and CT, which have focused on the differentiation of muscle-invasive and non-muscle-invasive BCa based on radiomic features [8-11]. To the best of our knowledge, this is the first study to predict muscle invasion based on radiomics shape features.

In a meta-analysis evaluating eight studies and 860 patients, the sensitivity and specificity of radiomic features in predicting muscle invasion were 82% (95%CI: 77-86%) and 81% (95%CI: 76-85%), respectively [8]. In these studies, all of the radiomics features, including the gray-level intensity features, were examined, and there was a wide range in the number of features (63-15,834), although the number decreased when feature-reduction methods were applied. In our study, we only considered shape features. Radiomics shape features derived from ADC maps had an accuracy of 83.3%, a sensitivity of 82.8%, and a specificity of 84%, while shape features derived from T2W images had an accuracy of 78.2%, a sensitivity of 82.7%, and a specificity of 73% within this dataset. The sensitivity and specificity of radiomics features in other studies using gray-level intensity distribution were similar to the sensitivity and specificity in our study using only shape features.

Volumetric shape features including volume, maximum 2D diameters, and 3D diameters and surface area were statistically different in the two imaging methods. However, some shape features obtained from T2W imaging, including maximum 2D diameter distances corresponding to axial and coronal images, were ineffective in terms of differentiation of invasion. Invasive tumors were flatter (plaque-like), and as a result, sphericity was higher in non-invasive tumors with both imaging modalities. Elongation and flatness were not statistically significant with either imaging modality. Overall, ADC-derived shape features had higher sensitivity, specificity, and accuracy than T2W imaging in the final machine learning algorithms. The results of this study are in line with the higher contrast resolution between the lesion and surrounding tissue in ADC mapping and thus should be considered as the DWI dominant sequence in multiparametric MR evaluation.

This study aimed to determine radiomic-only shape features to predict muscle-invasive BCa and to evaluate the diagnostic performance of this approach. Based on the use of radiomics shape features alone in BCa, we found that muscle-invasive tumors are less spherical, flatter, and have more volume in a larger area compared to non-muscle-invasive BCa using machine learning algorithms. Other texture features relevant to gray-level intensity distribution are highly affected by low-quality imaging between different scanners. Shape features are useful since they are less affected [12]. In addition, shape features can be used in daily practice through visual assessment without 3D segmentation, unlike other radiomics features [13]. The results of this study also support the use of machine learning algorithms in daily practice.

The study has several limitations. This was a single-center and single-scanner study, and thus future studies should confirm our results in larger populations. Since the VI-RADS imaging protocol started in our hospital in January 2020, we did not include older patients in the study, which limited the number of patients. However, it is valuable in that it is the first study conducted using shape features. 

## Conclusions

Shape features derived from T2W and ADC maps may be helpful for determining muscle invasion in BCa. Radiomic analysis based on the quantitative evaluation of geometric parameters has the potential to be used as a non-invasive test to predict muscle invasion in BCa. 
